# Promoting Maternal and Child Cardiovascular Health by Enhancing Skills and Education of Early-Stage Researchers

**DOI:** 10.1016/j.jacadv.2025.102043

**Published:** 2025-07-23

**Authors:** Charlotte A. Pratt, Stavroula K. Osganian, Ayesha Siddiqui, Debbie Vitalis, Candice Price, Alison G.M. Brown, Jonathan Moyer, Nicole Redmond

**Affiliations:** a National Heart, Lung, and Blood Institute, Bethesda, Maryland, USA; b National Institute of Diabetes and Digestive and Kidney Diseases, Bethesda, Maryland, USA; c Office of Disease Prevention, Bethesda, Maryland, USA

**Keywords:** cardiovascular health, early stage investigators, maternal and child

Cardiovascular disease (CVD) is the leading cause of death globally^1^ and the prevalence is high and rising in the United States. Among U.S. adults, aged 45 to 54 years, 55% of males and 48% of females have hypertension.^2^ Risk of CVD for women also changes throughout the life course, with increasing risk influenced by hormonal changes such as those during menarche and menopause as well as by adverse pregnancy outcomes.^3^, ^4^, ^5^, ^6^, ^7^ The United States has the highest rate of maternal morbidity and mortality among high-income countries, driven in part by significant disparities in maternal cardiovascular health (CVH).^1^ Data suggest that approximately 80% of excess maternal deaths are preventable^6^ and are primarily due to quality of care, lack of knowledge about warning signs, and maternal health behaviors.

National Heart, Lung, and Blood Institute (NHLBI) has made significant investments in maternal health research including the CHAP (Chronic Hypertension and Pregnancy) trial, the REBIRTH (Randomized Evaluation of Bromocriptine In Myocardial Recovery THerapy for Peripartum Cardiomyopathy), and the nuMoM2b (Nulliparous Pregnancy Outcomes Study: Monitoring Mothers-to-be Heart Health) studies.^5^^,^^6^ In 2020, the NHLBI launched the Maternal Health Community Implementation Program. The objective of Maternal Health Community Implementation Program is to test implementation strategies for the adoption and scaling of evidence-based interventions aimed at improving maternal health, especially in high-need communities.^7^^,^^8^ Building on prior research, in 2021, NHLBI developed a Funding Opportunity Announcement for the ENRICH (Early Intervention to Promote CVH of Mothers and Children) program. ENRICH aimed to test the effectiveness of an implementation-ready intervention designed to promote CVH and address CVH disparities in both mothers and young children who are of low socioeconomic status, live in low-resource rural or urban communities, or who are in different geographic regions of the United States with high burden of CVD risk factors. Preparing the next generation of researchers in maternal and child health is also a priority for NHLBI. The institute invests significant resources in skills development research training of early-stage investigators (ESIs) to ultimately prepare them to design and conduct rigorous, reproducible, and impactful research. Since FY 2009, NHLBI has established a payline of 10 percentile points above that for established investigators for Research Project Grants (R01).^9^

The traditional approach to training has focused on providing one-on-one mentorship with one or two primary mentors in a focused area of scientific expertise. With mentor oversight, the trainee designs and leads small-scale studies to prepare for independence. Although the trainee has substantial dedicated effort during the award, senior investigators are not always funded to serve as mentors and thus do not necessarily have dedicated time to devote to the trainees.

Furthermore, the small pilot project that a trainee proposes may not be considered to have yielded adequate preliminary data to support a larger scale investigator-initiated R01 grant application. These challenges are particularly an issue in community intervention trials which are more complex and difficult to design and implement as they require more than one cluster, group randomization, and larger sample sizes to account for correlations of participants within the same groups as well as approaches to avoid contamination. Multidisciplinary teams with various areas of content expertise are needed, for example, to design and implement complex, multicomponent obesity interventions.^8^ A traditionally focused short-term training experience is often not sufficient to face the real-world challenges and complexities of designing and conducting research in community settings, and additional advanced training and education are needed. Research training and education embedded in a community-based multicenter trial such as ENRICH offers a unique and robust training and educational experience for ESIs that overcomes some of these limitations and provides the opportunity to enhance valuable professional and technical skills gained through participation in a multisite study.

This article highlights the ENRICH trial and discusses NHLBI investment in ESIs to prepare the next generation of researchers in early CVH, with the goal of providing skills training and knowledge in clinical trial research, behavioral interventions, and implementation science to address maternal and child CVH.

ENRICH is a multicenter, community-based clinical trial comprised of 7 academic clinical and community centers located across the United States with multidisciplinary teams that include obstetricians, pediatricians, nutritionists, clinical psychologists, behavioral scientists, and statisticians as well as a single Data Coordinating Center. The overall goal of ENRICH is to evaluate the effectiveness of an implementation ready intervention delivered in the context of home visiting (HV) to improve CVH-related behaviors in pregnant women and their offspring.

The 7 ENRICH Clinical Centers partner with about 91 HV agencies and 125 supervisors and is expected to enroll about 3,300 mothers (6,600 parent/child dyads). The ENRICH governance structure includes a steering committee that is comprised of the clinical center principal investigators and an external chair and serves as the decision-making body for the trial. Several subcommittees including intervention, measurement, data and analysis, publications and ancillary studies, recruitment, retention and intervention implementation, and ESIs are included in the ENRICH Committee Structure. Ad hoc training groups are formed as needed. These teams are charged with developing various aspects of the protocol, study design, intervention, and data collection and overseeing development of ancillary studies and dissemination of results through publications and presentations.

## Early-stage investigators in ENRICH

The ESI subcommittee, which is unique to ENRICH, is charged with activities that foster training and career development of ESIs focused on improving early CVH, promoting CVH across the life course, and reducing CVH disparities from a multidisciplinary perspective. These investigators also actively participate in subcommittees and collaborate in decision-making, taking into consideration what will ultimately be feasible in the real-world setting of multiple HV programs while yielding valid and reliable results.

An example of such decisions related to study design and level of randomization—individual, HV professional, HV supervisor, or HV agency. Issues related to study power and contamination were discussed at length. Randomization at higher levels using a cluster randomized trial design would reduce the risk of contamination. However, effective sample size would likely be low due to correlated outcomes arising from similarities between cluster members. Failing to account for this correlation can result in inflated type I error rate.

Randomizing HV professionals would provide the greatest effective sample size, but contamination concerns remained as HV professionals were likely to interact with each other directly or indirectly via supervisors. Ultimately, supervisors were selected as the unit of randomization that could yield adequate power and minimal contamination. The discussions on the statistical approach were instructive to ENRICH investigators, including ESIs.

The advantages of training and education for ESIs in the context of ENRICH are several-fold.^9^^,^^10^ They have an opportunity to work with multiple experienced investigators and leaders in the field from various disciplines and different geographic locations who have dedicated effort on the study. Mentorship and training are thus essentially seamlessly integrated within the work of the subcommittees and research teams as they design and implement a rigorous common protocol with established milestones and deadlines. In addition, ESIs have several opportunities to network with established investigators as well as peers, thus fostering peer mentorship. During these experiences, ESIs benefit from listening to multiple perspectives and observing problem solving and leadership styles that promote cooperation and productive collaboration. Given the large sample across multiple sites and rigorous methods, ENRICH has the potential to generate high-quality data and results that are generalizable and may inform clinical and public health practice change. ESIs thus have an opportunity to publish these findings in high-impact peer-reviewed journals, as well as opportunities for proposing and leading ancillary studies and substudies that may be more competitive for funding of future projects. As an example, NHLBI supported the Childhood Obesity Prevention and Treatment Research in 2010 to 2017 that included early-stage investigators as members of the consortium.^10^ In this trial, 19 ESIs launched multiple scientific careers during program implementation: 6 were promoted, 22 manuscripts were published, 9 received NIH grants, and more than 95% were involved in scientific discoveries (eg, 14 were in academia and 4 worked with industry). These outcomes will also be tracked in the cohort of ESIs participating in ENRICH to assess the success of this model. Ultimately, ENRICH has the potential to be an efficient and effective approach to enhancing the training, education, and professional skills of ESIs. By producing a cadre of investigators who are better equipped to secure future funding, design, and conduct complex community-based real-world research. ENRICH has the potential to improve the future CVH of mothers and children.

## Summary

ENRICH provides an opportunity to train and mentor the next generation of maternal and child health researchers to promote CVH by providing skills training and promoting the transfer of knowledge and research skills from one generation to another.

## Funding support and author disclosures

ENRICH is supported by the 10.13039/100000050National Heart, Lung, and Blood Institute, under Data Coordinating Center U24HL163114 and 7 Clinical Centers: UH3HL162967, UH3HL162970, UH3HL162971, UH3HL162973, UH3HL163116, UH3HL163121, UH3HL163508. Collaborators include the National Institute of Diabetes and Digestive and Kidney Diseases, National Institute on Minority Health and Health Disparities, Office of Behavioral and Social Sciences Research, Office of Disease Prevention, Office of Research on Women’s Health, the Administration for Children and Families, and the Health Resources and Services Administration. The views expressed in this manuscript are those of the authors and do not necessarily represent the views of the National Heart, Lung, and Blood Institute, the National Institute of Diabetes and Digestive and Kidney Diseases, Office of Disease Prevention, the National Institutes of Health, or the U.S. Department of Health and Human Services. The authors have reported that they have no relationships relevant to the contents of this paper to disclose.
